# The Position of the Medical Examiner in Life Assurance.—I

**Published:** 1892-06-18

**Authors:** 


### The Practitioner's Mirror and Retrospect.

[*The Editor will be glad to receive offers of co-operation and contributions from members of the profession. All letters should be addressed to* The Editor, The Lodge, Porchester Square, London, W.]

### MEDICINE.

### THE POSITION OF THE MEDICAL EXAMINER IN LIFE ASSURANCE.—I.

As the necessity or even desirability of requiring a medical examination of every candidate for life assurance is being mooted in some of the leading British offices, it may be well to review the question in its various aspects, for there can be no doubt that medical examinations properly and skilfully conducted should be so valuable in the selection of lives as to be an altogether indispensable feature in life assurance work. In a recent address to the members of the Bristol Insurance Institute, Dr. Watson Williams remarked that first one office, then another, offers life policies without a medical examination, and probably many a representative has asked himself what is the good of the medical examinations, which have caused so many candidates to decline or be declined the advantages of life assurance. When we hear that Mr. Sutton, the President of the Institute of Actuaries, supposes that the medical examination "deters thousands of persons, of quite as good health really as those who propose, from proposing at all" we are inclined to agree with the editor of the *Review*, when he says that "it certainly does not follow that the examination should be done away with, but it does follow that Mr. Sutton's thousands upon thousands—however many that may be—require to be told that, as they are of good health, really they have nothing whatever to fear from the medical examination." In the early days of life assurance practically no medical examination was required, and so little value was attached to it that the offices were content with an informal reference to the medical attendant, for which they paid no fee. But very soon it was ascertained that if all were to be admitted to the office on the same conditions, it was absolutely necessary that some efficient mode of selecting lives should be adopted, and the medical examination become more of a reality. More extended experience has shown where the weak points of the examination were, and a more exact knowledge of medicine and more precise methods have rendered the examination more searching. Now it may be said that the medical examiner has failed to give exact results, but the same may be said of the actuary, for there is no doubt that in the past the premiums demanded were excessive, and were higher than experience has shown were necessary to cover the risks. Formerly risks were calculated on the Northampton table; more recently on the Carlisle table. But, in many companies, the still more exacting H.M. tables of the Institute of Actuaries are taken as the basis of the actuarial calculations, or the combined H.M. and H.M. 5 tables. What has brought about this repeated change in the basis of calculation? How is it that the leading companies are enabled to safely adopt these tables which give greater average expectation of life? It is the success of the medical selection of lives, for if the medical examinations had failed, the expectation of assured lives would remain the same as that of the whole population, whereas the calculations of the actuaries, and, therefore, the stability of the companies, directly depends on the fact that the average expectation of accepted lives will exceed that of the population generally.

The lecturer referred to the modified policies offered by some offices in which a declaration of health by the assuree was accepted in lieu of a medical examination. But, he asked, what good life would pay increased premiums on the chance of surviving his selected age and thus securing a compensation advantage? A bad life would hardly be accepted unless his declaration was misleading, and in making his declaration the candidate takes upon himself great responsibility and opens the way for litigation after death. What every sensible man wants nowadays, and will have is an indisputable policy, one that once granted can never be disputed on any ground whatever, whether death be due to natural causes, suicide, or foreign residence; and very soon every business man will know what kind of policy to go in for without the aid of the insurance agent. The medical examiner holds a most responsible position, for it is not by any means an easy matter, and requires considerable technical knowledge to decide at a single interview and examination whether a person is likely to live out his normal expectation, but this is a question which the life assurance examiner is called upon to do continually, and his decisions often involve his offices in great financial responsibilities, sometimes amounting to ten, twenty, or even a hundred thousand pounds. The greatest tact should be observed in the examination, for while it is essential that it should be sufficiently thorough, it is quite possible to be too thorough. Many candidates are extremely nervous, and a few minutes, quiet chat will generally put them completely at their ease, and show them at their best, while the want of tact and a clumsily conducted examination may send up the pulse and respiration rate and cause abnormal sounds in the heart, and thus, undoubtedly, many good lives have been rejected or only accepted at second-class rates. On the other hand, there are candidates who, knowing or suspecting some weak point in their physical state or family history, are purposely misleading; and in these cases a careless examination or a want of technical knowledge lands the office with unfair risks. Without traversing the whole ground we may illustrate the force of these remarks in regard to one or two of the points on which every examiner has to enquire. Firstly there is the family history, as indicating the pathological tendencies, the inherited weaknesses of the assuree. It is the duty of the examiner not to accept the statements of the candidate without strict investigation of all doubtful points. Thus child-birth is frequently given as the cause of death, when further enquiry shows that death took place months after parturition from a totally different cause, or that the deceased relative had had lung disease for some time before the birth of the child, and that death was really due to the lung affection. Again, inflammation or some other acute lung disease is often stated as the cause of death when further questions reveal that the disease was chronic and probably tubercular; on the other hand, it may be found that the candidate has given consumption, when the cause of death was acute inflammatory disease, or rheumatic fever, when the real cause was pœymia; or has in some way or other made his family or personal history tell against him unjustly. The skilled examiner will take care that a good life does not, in this way, unwittingly prejudice his own case for acceptance. It cannot be too strongly urged that attention should be directed to ascertain the truth as to the habits of the candidate, and this more especially in relation to alcohol. People's ideas as to what is a strictly temperate indulgence vary so widely that it is always necessary to make careful investigation of this point, and it need hardly be added that such investigation requires consummate tact. Loose, vague expressions and answers to questions are altogether useless.

A candidate who, while not having an altogether sound constitution, is perfectly steady in his habits, and leads a healthy life, is far more likely to exceed the average expectation of life than a strong and healthy individual whose manner of living will not bear inspection, and who indulges to excess in the pleasures of the table. The general appearance and bearing should be noted, and whether the real age corresponds with the apparent age; a prematurely aged and worn appearance, and early baldness, indicate a tendency to early degeneration and shortening of life.

*(To be continued.)*

